# Human Achilles tendon mechanical behavior is more strongly related to collagen disorganization than advanced glycation end-products content

**DOI:** 10.1038/s41598-021-03574-4

**Published:** 2021-12-17

**Authors:** Jennifer A. Zellers, Jeremy D. Eekhoff, Remy E. Walk, Mary K. Hastings, Simon Y. Tang, Spencer P. Lake

**Affiliations:** 1 Program in Physical Therapy, Washington University School of Medicine in St. Louis, 4444 Forest Park Ave, St. Louis, MO 63108 USA; 2 Department of Biomedical Engineering, Washington University in St. Louis, 1 Brookings Drive, St. Louis, MO 63130 USA; 3 Department of Orthopaedic Surgery, Washington University School of Medicine in St. Louis, 425 S. Euclid Ave, St. Louis, MO 63110 USA; 4 Department of Mechanical Engineering and Materials Science, Washington University in St. Louis, 1 Brookings Drive, St. Louis, MO 63130 USA

**Keywords:** Tendons, Metabolic disorders

## Abstract

Diabetes is associated with impaired tendon homeostasis and subsequent tendon dysfunction, but the mechanisms underlying these associations is unclear. Advanced glycation end-products (AGEs) accumulate with diabetes and have been suggested to alter tendon function. In vivo imaging in humans has suggested collagen disorganization is more frequent in individuals with diabetes, which could also impair tendon mechanical function. The purpose of this study was to examine relationships between tendon tensile mechanics in human Achilles tendon with accumulation of advanced glycation end-products and collagen disorganization. Achilles tendon specimens (n = 16) were collected from individuals undergoing lower extremity amputation or from autopsy. Tendons were tensile tested with simultaneous quantitative polarized light imaging to assess collagen organization, after which AGEs content was assessed using a fluorescence assay. Moderate to strong relationships were observed between measures of collagen organization and tendon tensile mechanics (range of correlation coefficients: 0.570–0.727), whereas no statistically significant relationships were observed between AGEs content and mechanical parameters (range of correlation coefficients: 0.020–0.210). Results suggest that the relationship between AGEs content and tendon tensile mechanics may be masked by multifactorial collagen disorganization at larger length scales (i.e., the fascicle level).

## Introduction

Diabetes affects 463 million people worldwide^1^ and is a leading cause of disability globally^2^. Characterized by impaired glycemic control, diabetes is associated with a three times greater risk of tendon injury^3^, and stiffening and functional shortening of the Achilles tendon is reported to contribute to abnormal walking mechanics, plantar ulceration, and limb loss^4–8^. Accumulation of advanced glycation end-products (AGEs) is one of the putative mechanisms for adverse diabetes-related changes in Achilles tendon^9,10^. AGEs form as a result of Amadori rearrangement between sugars and protein residues in connective tissues, accumulating with aging and pathologies with glycemic dysregulation, such as diabetes^11^. In tendon tissue, AGEs accumulation results in non-enzymatic binding of the collagen components, impairing molecular and fibrillar sliding of collagen^12,13^. Similar to what is observed in other tissues^14^, limitations in collagen sliding are reported to result in stiffening of the tissue^13^.

Attributing abnormal foot and ankle mechanics to diabetes-related stiffening and shortening of the Achilles tendon has made this tendon a target for intervention. For example, Achilles tendon lengthening surgery is one means of addressing presumed Achilles tendon dysfunction in the treatment of non-healing plantar wounds^15–17^. Despite positive outcomes associated with this treatment approach, the results of in vivo studies investigating diabetes-related changes in Achilles tendon stiffness are conflicting^9,18,19^. Studies in animal models of diabetic tendon have reported contradictory findings relative to the effect of diabetes on tendon stiffness^20–25^ and linear modulus^12,13,20,21,24–30^. The only study that has evaluated the effect of diabetes on ex vivo human tendon mechanics found a 21% reduction in Achilles tendon linear modulus in individuals with diabetes^31^; however, the limited amount of overall data on this topic lack consistency.

AGEs accumulation may contribute to chronic homeostatic dysregulation within the tendon, which is characterized by upregulation of inflammatory cytokines^32^, vascular changes^32^, altered cellular metabolism and autophagy^33,34^, and loss of collagen organization^35–38^. A combination of downstream effects of AGEs accumulation along with a variety of other factors could contribute to the disorganization of tendon fascicles that has been described in people with diabetes, particularly with type 2 diabetes^35–37^. Relationships between collagen organization and tensile mechanics have been well-established in tendon tissue^39–41^. Whereas impaired collagen sliding increases tendon stiffness^13^, collagen disorganization decreases tendon stiffness. As an example, unorganized collagen reduces tendon stiffness in degenerative conditions like tendinosis^41–45^.

The multiple effects of AGEs accumulation combined with other systemic and behavioral factors present a more complicated picture of tendon dysfunction in diabetic individuals. We hypothesize that AGEs accumulation and collagen disorganization can contribute to mechanical deterioration of the tendon, though presumably the effects of these factors would have opposing effects on characteristics like tendon elastic modulus. The purpose of this study was to examine relationships between AGEs content and collagen alignment with tensile mechanics in human Achilles tendon. We hypothesized that both AGEs content and quantitative measures of collagen alignment are predictive of mechanical parameters (specifically, linear modulus and hysteresis). We were primarily interested in investigating tendon mechanics at larger length scales, due to current clinical care intervening on tendon function at the level of the whole tendon (i.e., Achilles tendon lengthening).

## Results

Achilles tendon specimens were obtained from individuals undergoing below knee amputation (n = 13) or from autopsy (n = 3). Participants (10 male, 6 female) were a mean(standard deviation) age of 54.7(8.2) years old at the time of specimen collection with a body mass index of 31.1(6.3) kg/m^2^. Reasons for amputation included Charcot deformity (n = 5), osteomyelitis/wound infection/other infection (n = 4), and trauma/complications following orthopaedic surgery (n = 4). For cadaveric tissue, the cause of death was ALS (n = 1), respiratory failure/cancer (n = 1), and unknown (n = 1). Ten participants (all donors undergoing amputation) had been diagnosed with either type 1 (n = 2) or type 2 (n = 8) diabetes. None of the participants were having surgery specifically due to tendon injury, and tendons did not have fusiform thickening (suggesting tendinosis) on visual inspection. There were no statistically significant differences between participants with and without diabetes regarding age (*p* = 0.505), height (*p* = 0.280), weight (*p* = -0.893), or body mass index (BMI; *p* = 0.568). There were no statistically significant relationships between primary variables of interest and age, BMI, or weight (Table 1).

**Table 1:** Correlation matrix for primary variables of interest and participant demographics.

	Age	BMI^#^	Weight
**Mechanical testing**			
Hysteresis (%)	− 0.40	0.049	0.366
Peak stress (MPa)	0.224	0.132	− 0.283
Equilibrium stress (MPa)	0.196	0.071	− 0.281
Percent relaxation (%)	0.035	− 0.110	0.338
Linear modulus (MPa)	0.183	0.082	− 0.059
**Quantitative polarized light imaging**			
AVG DoLP (min)	− 0.199	− 0.110	− 0.103
STD AoP (max)(°)	0.089	0.159	0.369

No relationships were statistically significant (*p* < 0.05); Values are Pearson correlation coefficient unless otherwise noted, ^#^Indicates Spearman’s rho; average degree of linear polarization (AVG DoLP), standard deviation angle of polarization (STD AoP), minimum (min), maximum (max)average degree of linear polarization (AVG DoLP), standard deviation angle of polarization (STD AoP), minimum (min), maximum (max).

### Tensile mechanics

**Figure 1:**
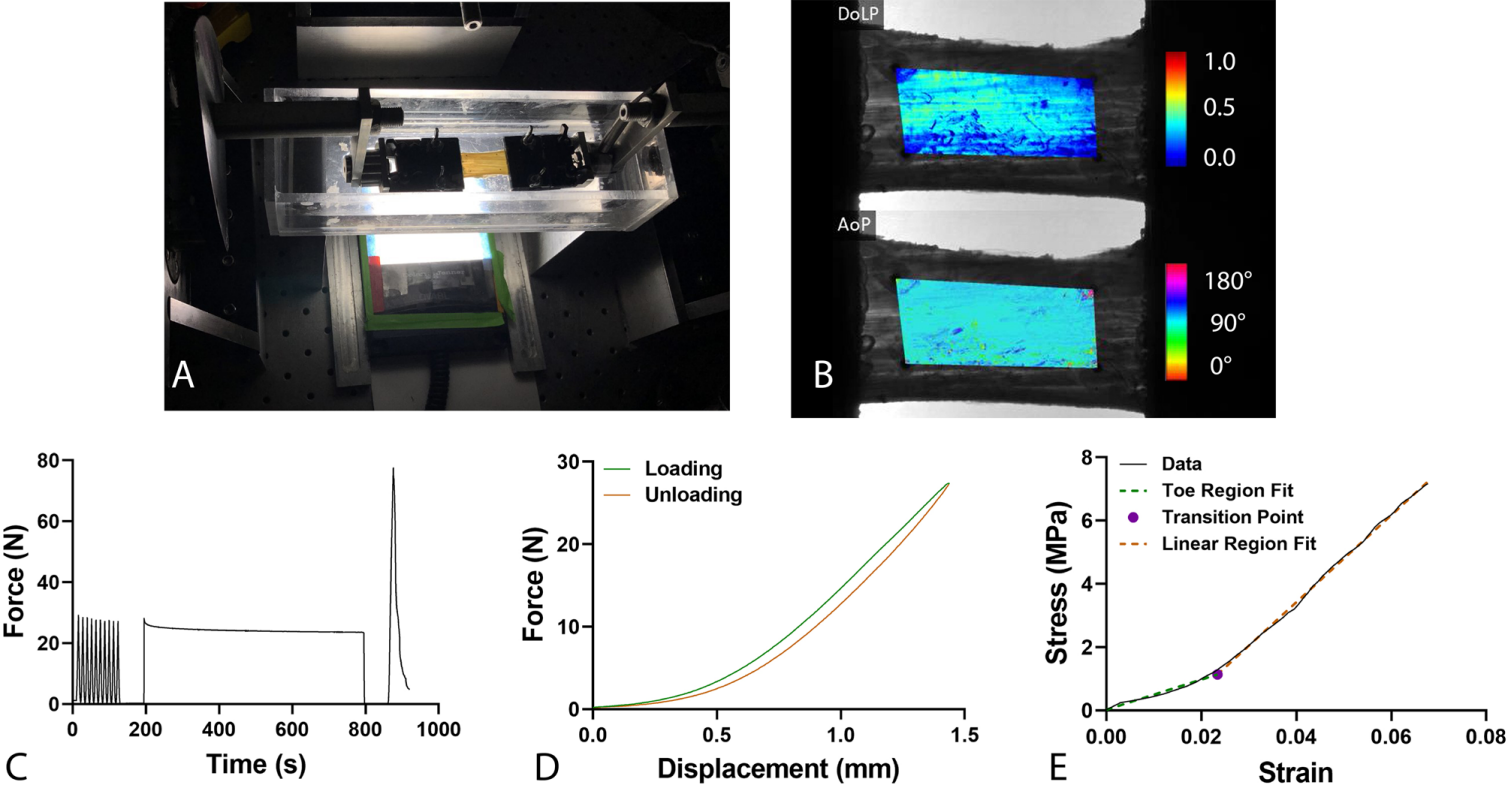
Set-up for mechanical testing and quantitative polarized light imaging (**A**) as well as representative data for a single tendon for degree of linear polarization (DoLP) and Angle of Polarization (AoP) on quantitative polarized light imaging (**B**). Representative mechanical testing data from the same tendon, including force over entire duration of tensile testing protocol (**C**), visualization of loading and unloading curves during 10th preconditioning cycle (used to calculate hysteresis; (**D**), and stress–strain curve showing bilinear curve fitting during ramp to failure (**E**).

Thinned specimens had a mean(SD) cross sectional area of 19.14(4.93) mm^2^. Specimens had a mean(SD) hysteresis of 9.56(3.28)% (Table 2). For stress relaxation testing, samples had a peak stress of 1.67(0.87) MPa, equilibrium stress of 1.33(0.78), and percent relaxation of 25.1(10.3)%. Samples had a linear modulus of 131.7(77.5) MPa. The surface features on two samples did not allow for strain tracking, and the linear modulus values were removed from analysis for these samples (both specimens were from donors with diabetes). There were no statistically significant differences in any mechanical testing variable between diabetic and non-diabetic specimens (Table 2). The experimental set-up for mechanical testing and representative data are shown in Fig. 1.

**Table 2:** Descriptive statistics for mechanical testing, collagen alignment, and advanced glycation end-products (AGEs) content.

	All specimens (n = 16)	Diabetes (n = 10)	Non-diabetes (n = 6)	Effect size(95% CI)
**Mechanical testing**				
Hysteresis (%)	9.56 (3.28)	9.29 (2.78)	10.01 (4.23)	0.201 (− 0.762–1.157)
Peak stress (MPa)	1.67 (0.87)	1.63 (0.81)	1.74 (1.03)	0.121 (− 0.839–1.077)
Equilibrium stress (MPa)	1.33 (0.78)	1.29 (0.74)	1.39 (0.93)	0.120 (− 0.840–1.075)
Percent relaxation (%)	25.1 (10.3)	25.2 (10.7)	25.0 (10.7)	− 0.017 (− 0.973–0.940)
Linear modulus (MPa)	131.7 (77.5)	131.1 (68.7)	132.4 (94.9)	0.015 (− 0.976–1.006)
**Quantitative polarized light imaging**				
AVG DoLP (min)	0.181 (0.074)	0.179 (0.083)	0.184 (0.066)	0.052 (− 0.906–1.008)
STD AoP (max) (°)	12.6 (7.6)	13.1 (9.4)	11.7 (3.4)	− 0.164 (− 1.119–0.798)
AVG DoLP (max)	0.261 (0.084)	0.268 (0.100)	0.247 (0.058)	− 0.232 (− 1.188–0.733)
STD AoP (min) (°)	9.1 (5.1)	9.2 (6.3)	8.8 (2.3)	− 0.077 (− 1.033–0.882)
AGEs content (units/collagen)	0.017 (0.007)	0.017 (0.007)	0.016 (0.005)	− 0.156 (− 1.112–0.805)

Values for diabetes and non-diabetes specimens are Mean(SD); average degree of linear polarization (AVG DoLP), standard deviation angle of polarization (STD AoP), minimum (min), maximum (max).

### Collagen alignment

When collagen was least aligned (occurring at zero strain) during the ramp to failure test, specimens had a mean(SD) degree of linear polarization averaged over the region of interest (AVG DoLP) of 0.181(0.074) (Table 2). The standard deviation of angle of polarization over the region of interest (STD AoP) is 12.6(7.6)°. When collagen was most aligned (occurring in the linear region), specimens had a AVG DoLP of 0.261(0.084) and STD AoP of 9.1(5.1)°. There were no statistically significant differences in collagen alignment between diabetic and non-diabetic specimens (Table 2).

### Advanced glycation end-products

AGEs content was a mean(SD) of 0.017(0.007) units/collagen in all samples, and no statistically significant differences in AGEs content was observed between diabetic and non-diabetic specimens (Table 2).

### Relationships between tensile mechanics and AGEs content/collagen organization

**Figure 2:**
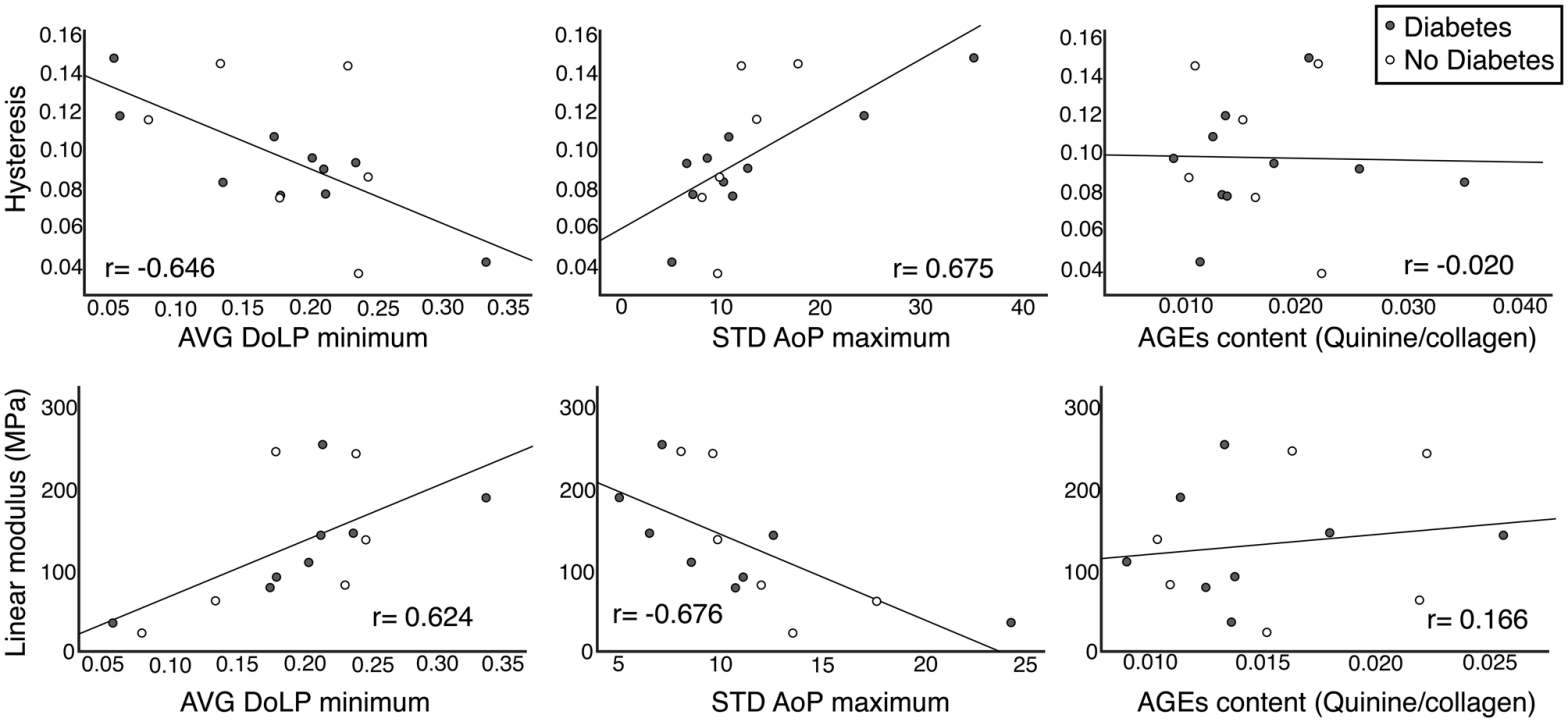
Scatterplots of relationships of tendon hysteresis (top row, hysteresis given as ratio) and linear modulus (bottom row) with collagen alignment [average degree of linear polarization (AVG DoLP) and standard deviation of angle of polarization (STD AoP)] and advanced glycation end-products (AGEs) content normalized to collagen content.

All of the assessed metrics of tendon tensile mechanics (linear modulus, hysteresis, peak stress, equilibrium stress, percent relaxation) had statistically significant relationships with all of the measures of collagen organization (AVG DoLP minimum/maximum, STD AoP minimum/maximum) (Table 3, Fig. 2). However, there were no statistically significant relationships between tendon tensile mechanics and AGEs content (Table 3, Fig. 2).

**Table 3:** Correlation matrix for relationship between measures of tendon tensile mechanics with collagen alignment and advanced glycation end-product (AGEs).

	AVG DoLP (min)	AVG DoLP (max)	STD AoP (min)^#^	STD AoP (max)	AGEs content
Linear modulus	0.624*	0.570*	− 0.727**	− 0.676**	0.166
Hysteresis	− 0.646**	− 0.608*	0.679**	0.675**	− 0.020
Peak stress	0.618*	0.604*	− 0.579*	− 0.699**	0.207
Equilibrium stress	0.601*	0.583*	− 0.626**	− 0.681**	0.210
Percent relaxation	− 0.698**	− 0.681**	0.700**	0.771**	− 0.057

*Indicates *p* < 0.05, **Indicates *p* < 0.01; Values are Pearson correlation coefficient unless otherwise noted, ^#^Indicates Spearman’s rho; advanced glycation end-products (AGEs), average degree of linear polarization (AVG DoLP), standard deviation angle of polarization (STD AoP), minimum (min), maximum (max).

## Discussion

We observed moderate to strong relationships between measures of collagen organization and tendon tensile mechanics, whereas no statistically significant relationships were observed between AGEs content and mechanical parameters. Relationships between AGEs content and tendon tensile mechanics may be present based on prior study findings^12,13^, but the results of this study suggest that collagen disorganization is the dominant mechanistic predictor of tensile behavior. While relationships between collagen organization and mechanical behavior have been previously described in other cadaveric tissues (e.g., supraspinatus tendon)^40,41^, this is the first study to characterize these relationships in human Achilles tendon where medical history (including diabetes status) was known. Ex vivo human Achilles tendon specimens are a model that allows for the appreciation of chronic glycation-related changes and recognizes the complexity of long-term systemic diabetes.

Studies investigating the isolated effects of non-enzymatic glycation have found alterations in collagen structure at multiple length scales of tendon architecture, impairing tendon mechanical behavior. Lee, et al.^46^, found tendons incubated in ribose solution demonstrated altered fibril nanostructure (diminished serial kinking of the fibril) after loading that was associated with impaired post-yield mechanics but not linear stiffness. A prior study of human Achilles tendon from individuals with diabetes^47^ found nanostructural changes similar to those described by Lee, et al.^46^ Studies by Fessel, et al.^12^, and Li, et al.^13^, have identified that non-enzymatic glycation limits sliding of tendon collagen at both the fibril and fiber levels; however, only impaired sliding at the fiber level translated to increased whole tendon stiffness. The techniques used to assess tendon mechanics in our study did not specifically isolate structural or mechanical properties of collagen fibrils and fibers. The lack of relationship between AGEs content and tensile mechanics (the combination of collagen mechanics at all levels of tendon architecture), however, joins a body of growing literature that questions long-standing assumptions regarding how AGEs-associated changes to fibril and fiber mechanics translate to altered mechanics at the fascicle and whole tendon levels^12,13,46,48^. It may be that AGEs play a more complex role in chronically placing abnormal strains on a tendon and initiating tendon degenerative changes—along with a biological role in elevating inflammation—that would not have been captured in this study given the limitations of a cross-sectional study design.

The effects of AGEs are not limited purely to increases in collagen cross-linking. AGEs accumulation has been found to drive disorganization at protein-level scales in other musculoskeletal tissues^49^. In the context of chronic AGEs accumulation, impaired cell function/signaling due to altered collagen sliding/mechanotransduction combined with a pro-inflammatory systemic environment may lead to degenerative tendon changes characterized by collagen disorganization^50^. It is possible that the collagen disorganization observed in this study could have been driven by a combination of the multiple effects of AGEs accumulation in collagenous tissue. In addition to the effects of AGEs accumulation, a host of other factors—including physical activity level^51^, dyslipidemia^52,53^, and obesity/high BMI^54,55^—can all contribute to collagen disorganization. The findings of this study align well with prior studies supporting the relevance of collagen organization on tissue tensile mechanical behavior^39–41^. From a clinical standpoint, addressing modifiable factors (like physical activity level, for example) may help to slow tendon degeneration and potentially improve collagen organization^45,56^, which would likely result in improved tensile mechanical behavior and could improve patient outcomes.

Clinical studies in individuals with diabetes have reported collagen disorganization^35,36^, but the implications of diabetes on in vivo tendon mechanics are less clear. Studies using ultrasound elastography have identified a reduction in tendon elasticity in individuals with diabetes, particularly when combined with peripheral neuropathy^18,19^. The findings of the present study in combination with studies supporting muscle fibrosis and atrophy in individuals with diabetes^57^ point to the need to better understand tendon dysfunction in people with diabetes to appreciate the complexity of this problem. A muscle with limited ability to place strain on the tendon (due to fibrosis, peripheral neuropathy, or chronic disuse/atrophy) may be unable to load the tendon into the linear region, and could explain why studies relying on volitional muscle contraction to quantify tendon mechanics (i.e. dynamometer with ultrasound) have reported alterations in tendon stiffness in individuals with diabetes^9^ that contradict tendon tensile behavior using techniques that do not require volitional muscle contraction^18,19,31^.

Direct comparisons of tendon mechanical properties and collagen organization are challenging due to differences in protocols and tendon models; however, data measured in this study were reasonably similar in magnitude to previously reported metrics. A prior study^58^ in human Achilles tendon with mean(SD) linear modulus of 346.8(68.1) MPa in individuals with diabetes and 414.0(66.5) MPa in individuals without diabetes, compared to 131.7(77.5) MPa for all tendons in the present study. Tendons in both studies were thinned, though this was done to different tendon dimensions, and tensile testing protocols differed, which may explain differences between studies. This is the first study to quantitatively measure human Achilles tendon collagen organization during loading conditions, however, the values observed in the present study are comparable to those observed in murine Achilles tendon in a prior study^59^.

Due to the challenges of procuring fresh human tissues, particularly as the Achilles tendon is often used as graft tissue, this study is of relatively small sample size which may limit its ability to detect weak relationships between tensile mechanics and glycation. Sample size also precluded more advanced statistical analyses and comparisons between specimens with and without diabetes. Additionally, it is important to consider that the individuals included in this study were undergoing lower extremity amputation, so loading on the evaluated tendon is likely less than in otherwise healthy individuals which could have implications for tendon mechanics and remodeling. It is possible that reduced collagen turnover, which was unable to be ascertained from this study, due to a lack of tendon loading resulted in tissues enriched in AGEs even in cases where the donor did not have diabetes, which could explain why there were was no clear differences in AGEs content between specimens from donors with and without clinical diabetes. A lack of heterogeneity in AGEs content amongst the included samples could have limited our ability to observe significant relationships between AGEs content and tendon tensile mechanical properties. Finally, factors such as sex have been inconsistently found to affect tendon mechanical properties in uninjured tendon in prior literature^60–62^, but we were not able to control for these factors in our analysis or perform subgroup analysis to compare differences between sexes due to the relatively small sample size.

In conclusion, the results of this study suggest that human Achilles tendon mechanical behavior is strongly related to collagen organization but does not scale with AGEs concentration. This is a clinically important distinction as there is some indication that mild collagen disorganization is potentially modifiable with rehabilitative treatment^56,63,64^, whereas the ability to effectively treat AGEs accumulation in tendon with currently available treatments is unknown. Future studies investigating temporal links between AGEs accumulation and collagen disorganization would be helpful in understanding to what extent AGEs accumulation chronically impairs tendon mechanics and biochemistry, contributing to tendon disorganization.

## Methods

### Study design and participants

Achilles tendon specimens were collected from individuals undergoing amputation. Individuals needed to be 18 years of age or older undergoing below knee amputation to be included. Exclusion criteria were diagnosed peripheral artery disease, known infectious diseases (human immunodeficiency virus, Hepatitis C, methicillin-resistant staphylococcus aureus, and vancomycin-resistant enterococci), or inability to obtain informed consent (for example, altered arousal/cognition due to trauma or pre-operative medications). In order to get a range of AGEs concentrations, individuals with and without diabetes were recruited for this study. This study was conducted with approval of the Washington University Institutional Review Board (IRB), was performed in accordance with the Declaration of Helsinki, and informed consent was obtained from all participants. Additional fresh, non-embalmed Achilles tendon specimens were obtained from a tissue procurement service through the autopsy department. Consent for collection/use of tissues for research was obtained from next of kin prior to autopsy. Specimens from deceased donors were deemed by the Washington University Human Rights Protection Office to not meet the federal definitions under the jurisdiction of an IRB and, therefore, falls outside the purview of the Human Rights Protections Office.

Tendons were collected via sharp dissection from the amputation site (a portion of the proximal Achilles tendon is retained during below knee amputation to form the gastrocsoleus flap) to just proximal to the insertion of the Achilles tendon on the calcaneus. After collection, all tendon specimens were stored in phosphate buffered saline (PBS)-soaked gauze at − 80 °C until mechanically tested. Tendons underwent three freeze–thaw cycles prior to mechanical testing. Age and diabetes status was confirmed for all participants/donors. Body mass index was recorded for participants undergoing amputation but was not available for donors of tendon tissue obtained from autopsy. Reason for amputation or cause of death was recorded for all participants/donors.

### Tendon tensile mechanics and collagen organization

Tendons were thinned only in the frontal (coronal) plane to 1 mm on a freezing-stage sliding microtome. Specimens were left to the native boundaries in the remaining planes. Cross-sectional area was measured using a non-contact laser scanning device (Keyence, LJ-V7080, Osaka, Japan)^59^. Thinned tendon specimens were clamped with sandpaper and positioned in a tensile testing rig (TestResources, 574LE2, Shakopee, MN). Tendons were submerged in PBS to ensure adequate hydration of the tissue. Aluminum strain beads (1/32″ in diameter) were adhered to the tissue with cyanoacrylate glue and used for optical strain tracking. Tendon samples were subjected to tensile testing: 0.1 MPa preload, 10 cycles of preconditioning at 6% strain, stress-relaxation at 6% strain for 10 min, and ramp to failure at a rate of 1% strain per second. One minute rest intervals were included between stress-relaxation and ramp to failure testing. Mechanical testing data were processed using a custom, automated Matlab code. The two-dimensional Lagrangian strain was calculated from the coordinates of the strain beads throughout the test. Loading curves followed the typical non-linear behavior of tendon. Hysteresis was calculated from the last pre-conditioning cycle as one minus the integral of the unloading curve divided by the integral of the loading curve. Peak and equilibrium stress as well as percent relaxation were calculated from stress relaxation, and linear modulus was determined using bilinear curve fitting of the ramp to failure test. Based on prior research emphasizing the relevance of tendon stiffness in individuals with diabetes^9^ along with pilot testing in other foot and ankle tendons by our lab group, the primary variables of interest from tensile testing were linear modulus and hysteresis.

Quantitative polarized light imaging (QPLI) was used during tensile testing to assess collagen organization at rest and with loading of the tendon^59,65^. The QPLI set-up includes a division-of-focal-plane polarization-sensitive digital camera^59^. QPLI leverages the birefringence of tendon tissue to quantitatively measure the strength and direction of collagen alignment, which are expressed for a region of interest (in this case, the tendon area within the strain-tracking beads) as the average degree of linear polarization (AVG DoLP) and standard deviation of angle of polarization (STD AoP), respectively. Higher DoLP represents more strongly aligned collagen, with a perfect linear polarizer demonstrating a value of 1. AoP represents the angle that the collagen is oriented, with 0° (or 180°) representing a perfect line running from clamp to clamp. Therefore, more organized tendon tissue has larger AVG DoLP (greater strength of alignment) and smaller STD AoP (indicating better uniformity of alignment) than less organized tendon tissue. QPLI data were recorded throughout tensile testing. For data reduction, the minimum and maximum AVG DoLP/STD AoP during the ramp to failure test were used for statistical analysis. On visual assessment of QPLI and tensile testing data, the minimum AVG DoLP/maximum STD AoP occurred at rest (representing the “least organized” collagen state) and the maximum AVG DoLP/minimum STD AoP occurred in the linear region of the stress–strain curve (representing the “most organized” collagen state). After mechanical testing, strain beads were removed from the tissue, and the tissue was wrapped in saline-soaked gauze and stored at − 80 °C for biochemistry.

### Quantification of advanced glycation end-products

AGEs were quantified using a fluorescence assay as previously described^66^. Specimens were papain digested and hydrolyzed. Fluorescence was compared to a quinine standard at an excitation of 370 nm and emission of 440 nm using a microplate reader. Collagen content was then assessed using a hydroxyproline assay^66^. AGEs concentration is reported as quinine normalized to collagen content.

### Statistical analysis

Descriptive statistics are reported as mean(sd) unless otherwise noted. Data were examined to ensure they met the assumptions of parametric statistical testing via the Shapiro–Wilk test and by viewing Q–Q plots. Relationships between AGEs content/collagen alignment on QPLI and tensile mechanics were tested using Pearson correlation coefficient. Body mass index and STD AoP did not meet parametric assumptions, so Spearman’s rho was used. This study was not powered for comparisons between specimens from individuals with or without diabetes; however, estimates of effect size may be helpful in the design of future studies. Therefore, we performed independent *t*-tests to compare findings between specimens from individuals with and without diabetes and have reported effect sizes (Hedges’ *g*)^67^ with 95% confidence intervals. Effect sizes (with cut-off) were interpreted as small (0.2), medium (0.5), and large (0.8)^68^. 95% confidence intervals crossing zero were considered not statistically significant^69^.

The datasets generated during the current study are available from the corresponding author on reasonable request.
